# Global Semantic-Sense Aggregation Network for Salient Object Detection in Remote Sensing Images

**DOI:** 10.3390/e26060445

**Published:** 2024-05-25

**Authors:** Hongli Li, Xuhui Chen, Wei Yang, Jian Huang, Kaimin Sun, Ying Wang, Andong Huang, Liye Mei

**Affiliations:** 1 School of Computer Science and Engineering, Wuhan Institute of Technology, Wuhan 430205, China; 2 Hubei Key Laboratory of Intelligent Robot, Wuhan Institute of Technology, Wuhan 430205, China; 3 School of Information Science and Engineering, Wuchang Shouyi University, Wuhan 430064, China; 4 State Key Laboratory of Information Engineering in Surveying, Mapping and Remote Sensing, Wuhan University, Wuhan 430079, China; 5 School of Computer Science, Hubei University of Technology, Wuhan 430068, China; 6 The Institute of Technological Sciences, Wuhan University, Wuhan 430072, China

**Keywords:** salient object detection, remote sensing image, semantic interaction, semantic perception, information entropy

## Abstract

Salient object detection (SOD) aims to accurately identify significant geographical objects in remote sensing images (RSI), providing reliable support and guidance for extensive geographical information analyses and decisions. However, SOD in RSI faces numerous challenges, including shadow interference, inter-class feature confusion, as well as unclear target edge contours. Therefore, we designed an effective Global Semantic-aware Aggregation Network (GSANet) to aggregate salient information in RSI. GSANet computes the information entropy of different regions, prioritizing areas with high information entropy as potential target regions, thereby achieving precise localization and semantic understanding of salient objects in remote sensing imagery. Specifically, we proposed a Semantic Detail Embedding Module (SDEM), which explores the potential connections among multi-level features, adaptively fusing shallow texture details with deep semantic features, efficiently aggregating the information entropy of salient regions, enhancing information content of salient targets. Additionally, we proposed a Semantic Perception Fusion Module (SPFM) to analyze map relationships between contextual information and local details, enhancing the perceptual capability for salient objects while suppressing irrelevant information entropy, thereby addressing the semantic dilution issue of salient objects during the up-sampling process. The experimental results on two publicly available datasets, ORSSD and EORSSD, demonstrated the outstanding performance of our method. The method achieved 93.91% S_α_, 98.36% E_ξ_, and 89.37% F_β_ on the EORSSD dataset.

## 1. Introduction

As an extension of the visual attention mechanism in target segmentation tasks, salient object detection (SOD) aims to accurately identify significant regions within a scene [1]. In the domain of SOD, it is often necessary to extract distinctive features from images to distinguish between targets and backgrounds. Information entropy can be used to measure the complexity and information content of different regions in an image. By calculating the information entropy of each pixel or region in the image, regions with high information entropy can be selected as potential target areas because targets typically introduce more information. By identifying high information entropy regions in the image, SOD can effectively discover key geographic features in remote sensing images (RSI), such as buildings, roads, water bodies, etc., providing reliable support and guidance for urban planning [2], resource management [3], environmental monitoring [4], and other fields. Therefore, by measuring the information content of different regions in the image, SOD technology can gain a deeper understanding of the distribution and structure of information in the image. This enables focusing attention on regions that may contain targets, thereby enhancing the efficiency and accuracy of SOD.

The application of SOD in RSI holds significant practical value for resource monitoring, urban planning, agricultural development, and other areas [5,6]. In terms of resource monitoring, SOD can help identify and monitor resources, such as water bodies, vegetation, and land, providing precise data support for resource management. For example, by detecting water bodies, it can realize the monitoring of water resources and analyze changes in water bodies, providing important references for the utilization and protection of water resources. In the aspect of urban planning, SOD contributes to identifying important buildings and transportation routes in cities, providing data support for urban planning and traffic management. In addition, SOD can also identify important targets, such as farmland and crops, analyze cultivation areas and crop types, and assist farmers in optimizing planting schemes, thereby improving crop yield and quality.

Unlike natural scenes [7,8], SOD faces numerous challenges in RSI. Firstly, RSI typically exhibit complex backgrounds and diverse distributions of objects, making accurate identification of salient objects more difficult. Secondly, RSI are susceptible to disturbances, such as atmospheric conditions and cloud cover, leading to decreased image quality and subsequently affecting the accuracy of SOD. Additionally, remote sensing images contain many small-scale objects, such as trees and vehicles, posing challenges to their identification and localization. Therefore, addressing these challenges requires comprehensive consideration of rich local features, acquiring global dependencies, and enhancing the semantic representation capability of salient objects.

Existing salient segmentation approaches primarily depend on CNN for feature extraction. These methods design multiple functional modules to explore the inherent relationships between features at various levels, enhancing contextual information. Zhou et al. [9] designed two decoder branches for multitask processing, simultaneously obtaining edge information and semantic features, using edge information to enhance the saliency of each level of features. Zheng et al. [10] enlarged the receptive field to capture more contextual information, enhancing the adaptability of the model to salient objects in different scenes. Liu et al. [11] explored the connections between foreground, edge, background, and global features, achieving feature complementation on a single-level feature map. However, they merely enhanced single-scale local features and ignored the potential connections among multi-scale features, which did not fully utilize the salient information in each level of features. Therefore, some researchers have tried to explore how to utilize contextual information of multi-level feature to enhance the understanding of salient objects. Gong et al. [12] proposed a strategy using semantic matching and edge alignment to achieve channel interaction between different features. Additionally, Li et al. [13] adopted a strategy of progressively refining features to generate abstract semantic features and guide the positioning of low-level features. Furthermore, Zeng et al. [14] aggregated global and local information by coordinating adjacent contextual information, thereby further improving the performance of the model.

However, convolutional networks are incapable of capturing extensive global information due to their limited receptive fields [15]. On the other hand, the transformer [16] is renowned for its ability to capture global dependencies between word vectors in NLP. Scholars have attempted to apply transformers to the computer vision domain. The Vision Transformer [17] segmented input images into small patches and transformed these patches into linear embeddings, serving as the input for transformers, marking the first application of transformers in the field of images. Liu [18] proposed a sliding-window mechanism to mitigate the computational complexity of transformers, restricting attention calculations to a window. The Pyramid Vision Transformer [19,20] introduced a feature pyramid into transformers, allowing them to perform various downstream dense prediction tasks. Xie [21] redesigned the encoder and decoder, adopting a hierarchical encoder structure to output multi-scale features and fusing them together in the decoder. Therefore, Gao [22] proposed an adaptive spatial tokenization transformer to extract features, gradually integrating contextual dependencies and addressing the issue of insufficient perception range. Zhang [23] designed a dual-stream encoder that simultaneously utilizes CNN and the transformer to extract features.

Undoubtedly, the research on these methods has provided valuable insights and inspiration for SOD. However, some issues remain unresolved. Firstly, some methods based on feature extraction with ResNet or VGG lack sufficient capability in feature extraction, failing to fully comprehend the semantic information of salient objects. Secondly, due to the obvious semantic disparities among features at different levels, simply fusing them may cause the loss of salient semantics, thereby failing to fully utilize salient information in multi-scale features. Lastly, some methods lack the capability to perceive small-scale targets, leading to the occurrence of omissions. Therefore, we design a Global Semantic-aware Aggregation Network to aggregate multi-scale salient information, achieving global–local semantic interaction and salient semantic feature perception. Specifically, to fully utilizing the advantages of convolution and self-attention, we adopt UniFormer to extract local features and global dependencies of salient objects. This not only preserves the detailed texture information and edge contours of salient objects but also enables the model to comprehend the overall information of the image, resulting in better handling of significant targets in complex scenes. Meanwhile, we propose a Semantic Detail Embedding Module (SDEM) to achieve feature fusion and semantic complementation by coordinating information across different scales, fully utilizing salient information in multi-scale features. Finally, considering the issue of semantic dilution of salient objects during the process of feature map restoration to the original image, we propose a Semantic Perception Fusion Module (SPFM) in the decoder to compensate for the lost salient features during up-sampling.

Our primary contributions are outlined as follows:

(1) We design a Global Semantic-aware Aggregation Network, named GSANet, to provide a crucial solution for precise localization and semantic understanding of salient objects in remote sensing images. This approach calculates the information entropy of each pixel in the image, measuring the complexity and information content in different regions, coordinating global and local semantic information, focusing on high-entropy areas, and deeply excavating the semantic features of salient objects.

(2) We propose a Semantic Detail Embedding Module (SDEM) for the fusion of multi-scale features and enhancement of semantic representation capabilities. SDEM explores potential connections between different scale feature maps, dynamically fusing shallow texture details and deep semantic features. Therefore, SDEM enhances the ability of the model to integrate salient information of different scales, efficiently aggregates the information entropy of salient regions, and improves the perception and understanding level of the model to salient objects in complex scenes.

(3) We propose a Semantic Perception Fusion Module (SPFM) to address the issue of semantic dilution of deep features during the up-sampling stage. SPFM regards salient semantic information as prior knowledge, enhancing the perception capabilities of each level’s feature map for the salient features.

The remaining structure of this paper is as follows. In Section 2, we describe the motivation and architecture of GSANet. In Section 3, we demonstrate the excellence of GSANet through experiments. In Section 4, we engage in discussion. Finally, in Section 5, we provide the conclusion and outlook.

## 2. Materials and Methods

### 2.1. Network Overview

**Figure 1:**
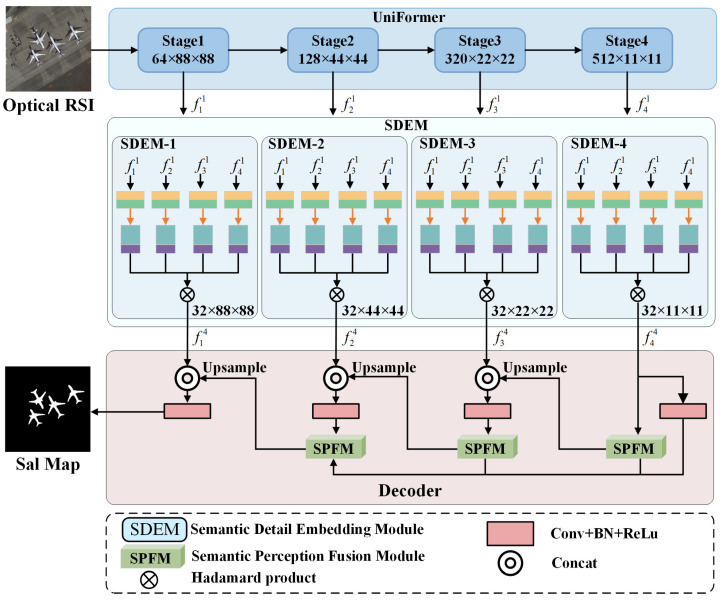
The overall framework of GSANet consists of three parts: the UniFormer, SDEM, and the decoder with SPFM. $f_{i}^{1}$ and $f_{i}^{4}$ (*i* = 1, 2, 3, 4), respectively, denote the multi-scale features extracted by the UniFormer and the features processed by the SDEM.

The challenges encountered in SOD from RSI include interference from shadows, the blurring of foreground and background information, and the omission of small-scale targets during the detection process. Therefore, we proposed a novel approach named GSANet that utilizes multi-level semantic fusion to fully exploit salient information, while simultaneously increasing the detection accuracy of salient objects through global semantic perception. The architecture of GSANet is shown in Figure 1. The network comprises three main components: the feature extraction module UniFormer [24], the Semantic Detail Embedding Module (SDEM), and the Semantic Perception Fusion Module (SPFM). UniFormer efficiently encodes input information, extracting abstract salient features while avoiding the generation of local redundancy. The SDEM aggregates four different levels of feature inputs, fully utilizing salient information in multi-scale features. The SPFM injects high sensitivity to semantic information during the decoding process, thereby enhancing the perception ability of the model to salient objects.

### 2.2. UniFormer

**Figure 2:**
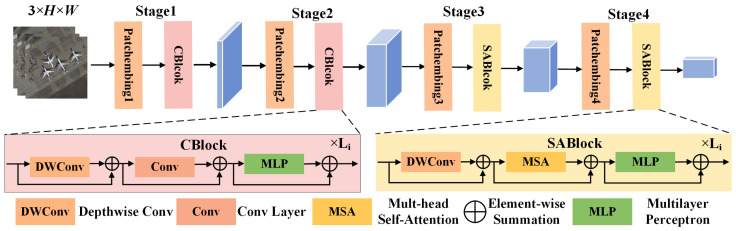
Illustration of the UniFormer structure. UniFormer adopts convolutional modules to extract shallow features and self-attention modules to extract deep features.

In the current methods, the main architectures for feature extraction include CNN and the transformer [25]. CNN, with convolution as its core operation, excels at extracting local details and effectively capturing local features and texture information in images. Nevertheless, CNN has limitations in capturing global dependencies, resulting in the model struggling to comprehend the correlation between different regions of the image. The transformer adopts self-attention to extract global dependencies, thereby facilitating the model to better comprehend the global semantic information within images. However, self-attention captures tokens with high similarity, leading to the occurrence of information redundancy in shallow networks, which negatively impacts the efficiency of the model learning process. It is necessary to find an effective way to combine the advantages of CNN and the transformer while addressing their individual limitations. Therefore, we adopted UniFormer [24] to extract salient features from remote sensing images, preserving more details in salient regions while avoiding irrelevant information from overshadowing salient features. The UniFormer architecture is illustrated in Figure 2.

In SOD, preserving the local details and capturing the global semantics of RSI are crucial. The local details encompass crucial information, such as the outlines of buildings, the textures of roads, and other subtle features, which are typically key characteristics of salient targets within the image. Meanwhile, the global semantics reflect the interrelation and overall structure among different regions within the image, such as the layout of urban areas and the distribution of vegetation. Therefore, we need a method to effectively balance the acquisition of local details and global semantics. The UniFormer module, with its staged design and integration of convolutional blocks and self-attention blocks, provides a solution to this balance issue. In stages 1 and 2, it utilizes convolutional blocks (CBlock) to extract local details, accurately capturing the small targets and subtle features in the image. In stages 3 and 4, it leverages self-attention blocks (SABlock) to effectively comprehend the global semantics of the image, thereby better grasping the overall structure and semantic information of the image. Each stage outputs a feature map, denoted as $f_{i}^{1}\in R^{C_{i}\times H_{i}\times W_{i}}\;(i=1,2,3,4)$, where C, H, and W are the channel, height, and width of the feature map, respectively.

Specifically, convolution adopts the mechanisms of local connectivity and weight sharing, excelling at capturing shallow texture information and local details. Local connectivity means each neuron is only connected to a small portion of the input data, enabling the network to focus more on detecting local features, such as edges and corners. Weight sharing enhances the generalization of the model, making it easier to capture common features in images, such as texture and shape. Self-attention is particularly effective at capturing global dependencies in images, primarily because it can simultaneously consider all positions within the image and effectively weight and aggregate them. This global consideration enables the model to comprehend long-range dependencies within images, thus better grasping the overall structure and semantics of the image.

### 2.3. Semantic Detail Embedding Module (SDEM)

In SOD, clear-edge structures and rich semantic features are considered effective means to enhance the pronounced disparity between the foreground and background. Clear-edge structures contribute to accurately capturing object contours, providing visual clues for the localization of salient objects. Meanwhile, rich semantic features provide valuable contextual information for salient objects, assisting the model in comprehensively understanding the connotations and background of salient targets [26]. In shallow neural networks, feature maps have higher spatial resolution and carry richer spatial positional information. However, they appear relatively vague in semantics. In contrast, feature maps in deep networks contain more abundant semantic information but lack spatial texture details. This indicates that shallow networks focus on capturing spatial fine-grained structures in images but have limited capacity for expressing abstract semantics. Deep networks concentrate more on extracting high-level semantic features but may lose some detailed expression of spatial texture in the process. As a consequence, how to integrate the strengths of shallow and deep features to overcome their respective limitations is a significant research challenge. On the other hand, RSI contain rich information, including not only a large number of small targets but also complex geographical features. However, due to the uneven distribution of this information in the images and its possible existence across different scales, it implies that a single feature often struggles to fully capture and express this information. Therefore, to fully utilize the abundant information in the images and enhance the perceptual capability of model, we need to consider features at different scales, comprehensively. By integrating multi-scale features, we can gain a more comprehensive understanding of the image content and enable the model to more effectively capture the relationship between targets and their environment, thereby improving the performance and accuracy of detection.

Inspired by [27], we proposed a Semantic Detail Embedding Module (SDEM) to fuse global dependencies, local texture details, spatial positional information, and deep semantic features, which enables the model to comprehensively understand and fully utilize salient information between multi-scale features. The SDEM weights the single feature with attention, enabling the model to focus more specifically on and emphasize the crucial feature information. In addition, the SDEM aggregates multi-scale information onto a single feature map using the Hadamard product, effectively achieving the fusion of multi-scale features. This approach not only preserves the spatial structure and semantic information of the original features but also ensures the consistency and integrity of multi-scale features.

**Figure 3:**
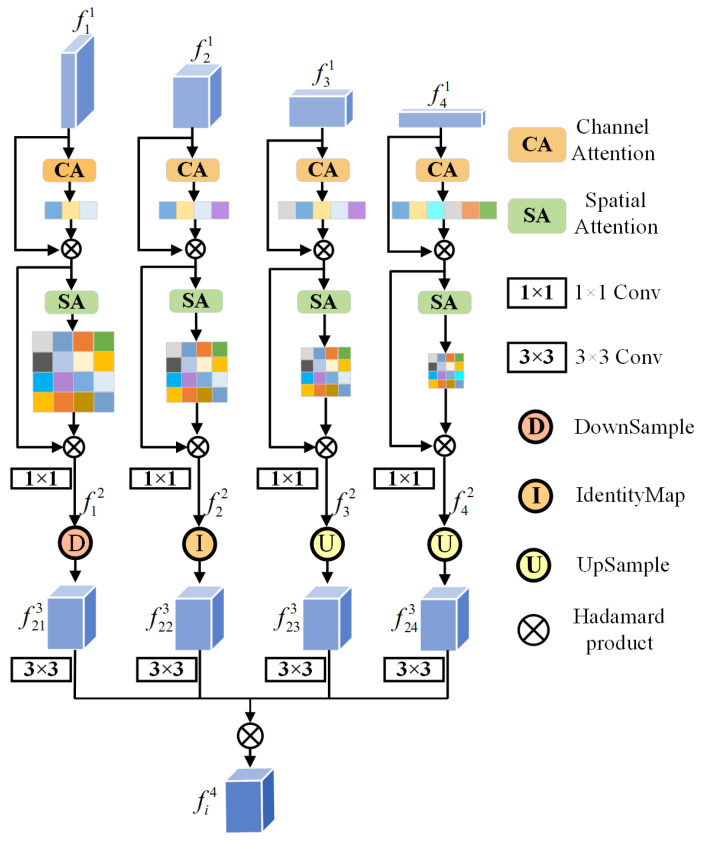
Illustration of the SDEM structure. As an example, we only demonstrated the processing of the feature map at the second level.

When dealing with complex scenes in RSI, the SDEM plays a crucial role. On the one hand, it embeds salient semantic into shallow features, enhancing the semantic representation capability of salient targets, thereby enabling the model to more accurately understand the semantic content of salient objects. On the other hand, the SDEM injects spatial texture details into high-level features, contributing to the precise localization of object edge contours, and enhancing the level of perception of key areas in complex scenes by the model. Therefore, the SDEM contributes to a more comprehensive analysis and understanding of images by fully leveraging semantic features and edge information. The structure of the SDEM is illustrated in Figure 3.

The SDEM fuses the shallow spatial texture structure and deep abstract semantic representation to fully exploit salient information in multi-scale features. Specifically, for the *i*-th SDEM, it receives four input feature maps from the encoder, denoted as $f_{i}^{1}\left(i=1,2,3,4\right)$. When processing the feature map at the *i*-th level, a weighted operation was first performed using channel attention and spatial attention mechanisms [28], assigning more weight to semantic features and boundary information of salient objects. This enhanced both local spatial details and global channel features of salient objects. After that, a convolution was performed to normalize the number of channels, adjusting it to 32. The detailed steps of the entire process are as follows:

(1) $$f_{i}^{2}=conv\left(SA\left(CA\left(f_{i}^{1}\right)\right)\right)$$

where $f_{i}^{1}$ denotes the *i*-th feature map, CA(·) is channel attention, SA(·) is spatial attention, and conv(·) is a 1 × 1 convolution used to adjust the channels.

Next, regarding the feature map $f_{i}^{2}$ as prior information, the resolution of the remaining feature maps, $f_{j}^{2}\;(j=1,2,3,4)$, was adjusted to align with $f_{i}^{2}$. The specific formula is as follows:

(2) $$f_{ij}^{3}=\left\{\begin{array}{ll} Down\left(f_{j}^{2}\right) & if\;j<i\\ Identity\left(f_{j}^{2}\right) & if\;j=i\\ Up\left(f_{j}^{2}\right) & if\;j>i \end{array}\right.$$

where Down, Identity, and Up, respectively, denote the operations to scale the resolution of the feature map $f_{j}^{2}$ to match that of $f_{i}^{2}$ using adaptive average pooling, identity mapping, and bilinear interpolation. The final output is denoted as $f_{ij}^{3}$.

Then, a 3 × 3 convolution was applied to all resampled feature maps. Through element-wise multiplication, the multi-scale feature information was gradually fused into the *i*-th feature map. Then, it can retain richer deep features and more detailed shallow information, resulting in the final output, denoted as $f_{i}^{4}$:

(3) $$f_{i}^{4}=Hadamard(\theta_{ij}(f_{ij}^{3}))$$

where Hadamard(·) denotes element-wise multiplication, and $\theta_{ij}$ is the parameters of the 3 × 3 convolution.

### 2.4. Semantic Perception Fusion Module (SPFM)

In the classical U-Net structure, the encoder extracts deep features, while the decoder restores high-level feature maps to the original image resolution through up-sampling. However, during the up-sampling process, the decoder faces two common issues. Firstly, the semantic information of deep features is gradually diluted during up-sampling. This leads to insufficient expression of semantic features, which causes fragmentation of edges and internal semantic voids in salient objects. Secondly, there exists a disparity in semantics among multi-scale feature maps. Simply combining them may result in semantic confusion between the foreground and background information, thereby interfering with the accurate identification of significant targets.

**Figure 4:**
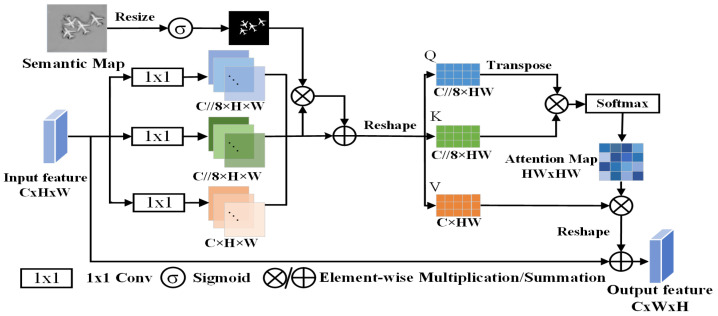
Illustration of the SPFM structure. The SPFM enhanced contextual awareness of salient objects through salient semantic maps.

Therefore, we introduced a Semantic Perception Fusion Module (SPFM) into the decoder, using the most semantically rich high-level feature map as prior knowledge to compensate the loss of salient semantics during the fusion process of multi-scale feature maps. The SPFM explored the mapping relationship between global features and local details through self-attention mechanisms, embedding global semantic information into local features. This enhanced the ability of model to perceive salient targets, enabling it to more effectively capture and retain salient semantic information. During the up-sampling process, the SPFM guided the decoder to fuse semantic information from different-level feature maps, allocating more attention weights to the semantic features of salient targets. This enabled the model to prioritize salient targets while disregarding irrelevant factors. The structure of the SPFM is illustrated in Figure 4.

Inspired by [29,30,31], the SPFM first utilized the salient semantic map as prior knowledge to weight the input information, and then employed self-attention to achieve context awareness, guiding the model to perceive the spatial distribution of salient objects. Through this process, SPFM was able to allocate weights for different regions of the image, injecting additional semantic information into the region of salient targets on the input feature map, and preserving this crucial information as much as possible.

Firstly, the semantic map was resized to align with the size of the input feature map. Subsequently, following processing through the sigmoid function, the value of each pixel in the semantic map was mapped to the range of 0–1, resulting in a semantic weighting map, $W_{S}$. In $W_{S}$, larger pixel values indicate increasing importance of information at the corresponding positions:

(4) $$\begin{array}{c} W_{s}=sigmoid\left(f_{s}\right) \end{array}$$

On the flip side, the input feature map underwent linear projection to generate three attention components, and these were further subjected to residual processing with the semantic weight map, $W_{S}$, ultimately producing the weighted attention components Q, K, and V. This operation, through fusing semantic information with attention components, elevated the weight proportion of pixels associated with salient targets. It led the model to effectively capture salient information in the input feature map during the process of encoding contextual information for input features through the interaction of three attention components (Q,K,V). The calculation process is as follows:

(5) $$\begin{array}{c} Q\\ K\\ V \end{array}=\left\{\begin{array}{c} (W_{s}+1)conv_{q}\left(f_{input}\right)\\ (W_{s}+1)conv_{k}\left(f_{input}\right)\\ (W_{s}+1)conv_{v}\left(f_{input}\right) \end{array}\right.$$

where $W_{S}$ denotes the semantic weight, $f_{input}$ denotes the input feature, and $conv_{q}$, $conv_{k}$, and $conv_{v}$ are, respectively, query convolution, key convolution, and value convolution of linear projections.

The components Q, K, and V were computed according to the following formula, resulting in the ultimate output:

(6) $$out=V\cdot Softmax\left(\frac{Q^{T}K}{\sqrt{d}}\right)+f_{input}$$

where d denotes the dimensionality of Q, K, and V.

### 2.5. Loss Function

We adopted a hybrid loss function to train the proposed network. This hybrid loss function comprises two components: Intersection Over Union (IOU) loss and Binary Cross-Entropy (BCE) loss. The IOU loss evaluates the precision of the model in predicting the position and shape of the target, while the BCE loss focuses more on the binary classification performance of model. By combining these two loss functions, the model can be comprehensively trained to achieve better performance in both target localization and target classification. The process for computing the overall loss is as follows:

(7) $$L_{total}=L_{IOU}\left(Sal,GT\right)+L_{BCE}\left(Sal,GT\right)$$

where $L_{IOU}$ and $L_{BCE}$ denote the IOU loss and BCE loss, respectively. GT is the ground truth label, and Sal is the salient prediction map.

## 3. Results

### 3.1. Experimental Setup

(1) Datasets: We conducted experiments on two datasets, ORSSD [32] and EORSSD [33]. The ORSSD dataset comprises 800 images with pixel-level annotations, divided into 600 images for training and 200 images for testing. These images are predominantly sourced from Google Earth, incorporating data from multiple satellites and aerial photography. Their spatial resolution ranges from 0.5 to 2 m. The EORSSD dataset is an extension of ORSSD, with an additional 1200 samples from Google Earth. It includes 2000 images with ground truth (GT), with 1400 images allocated for training and the remaining 600 for testing. Compared to ORSSD, EORSSD faces greater challenges in dealing with small targets, more complex scenes, and handling image interference. Additionally, we augmented the training data by performing rotations of 90°, 180°, and 270°, as well as horizontal flips followed by rotations with the same angles for both the images and the GT. Finally, the training samples for ORSSD and EORSSD reached 4800 and 11,200, respectively.

(2) Network implementation: All experiments were conducted using the advanced deep learning framework PyTorch [34], trained on a high-performance NVIDIA graphics processor 4060ti GPU (16 GB memory). During training, we utilized UniFormer [24] as the feature extraction network, initializing it by loading pre-trained weights. The resolution of input images and GT was adjusted to 352 × 352. We adopted the Adam optimizer for network training, with a batch size of 8, 45 epochs, and an initial learning rate set to 1 × 10^−4^. It is worth noting that after every 30 epochs, the learning rate decayed according to the specified schedule, reaching 1/10 of the initial value. The code is available at https://github.com/OrangeCat12352/GSANet.

### 3.2. Evaluation Metrics

To comprehensively evaluate the performance of the model, we adopted a series of common performance metrics for quantitative analysis. These metrics included S-measure ($S_{\alpha }$) [35], mean absolute error (MAE), E-measure ($E_{\xi }$) [36], F-measure ($F_{\beta }$) [37], PR curve, and F-measure curve.

The S-measure comprehensively considers both object similarity and region similarity, providing a comprehensive evaluation of the structural similarity between the detected results and the real objects. It is represented as:

(8) $$S_{\alpha }=\alpha \times S_{o}+\left(1-\alpha \right)\times S_{r}$$

where the weight coefficient α is set to 0.5, assigning equal weight to both object similarity $S_{o}$ and region similarity $S_{r}$.

MAE evaluates the mean absolute error between the prediction result (Sal) and the ground truth (GT). A smaller MAE indicates that the predicted results are closer to the ground truth. It can be expressed as follows:

(9) $$\begin{array}{c} MAE=\frac{1}{W\times H}\sum_{i=1}^{W}\sum_{j=1}^{H}\left|\mathrm{Sal}\left(i,j\right)-GT\left(i,j\right)\right| \end{array}$$

where W and H represent the width and height of the image, respectively, while (i,j) denotes the pixel coordinates.

The *E*-measure is an enhanced matching metric used to measure the matching degree between the global average and local pixels. The metric is defined as follows:

(10) $$\begin{array}{c} E_{\xi }=\frac{1}{W\times H}\sum_{i=1}^{W}\sum_{j=1}^{H}\xi_{s}\left(i,j\right) \end{array}$$

where $\xi_{s}$ is the enhanced alignment matrix, capturing two properties of the binary mapping: pixel-level matching and image-level statistics.

The F-measure combines *Precision* and *Recall*, serving as their weighted harmonic mean. It is a commonly used comprehensive evaluation metric and can be expressed as:

(11) $$\begin{array}{c} F_{\beta }=\frac{\left(1\;+\;\beta^{2}\right)\;\times \;Precison\;\times \;Recall}{\beta^{2}\;\times \;Precison\;+\;Recall} \end{array}$$

where β is the weight coefficient between Precison and Recall.

### 3.3. Comparison with State-of-the-Art

To validate the advancement of our approach, we selected 15 state-of-the-art SOD models for comparison, including CNN-based methods, such as SAMNet [38], HVPNet [39], DAFNet [33], MSCNet [40], MJRBM [41], PAFR [42], CorrNet [13], EMFINet [43], MCCNet [11], ACCoNet [14], AESINet [44], ERPNet [9], and ADSTNet [45], and transformer-based methods, such as HFANet [46] and GeleNet [47].

#### 3.3.1. Visual Comparison

**Figure 5:**
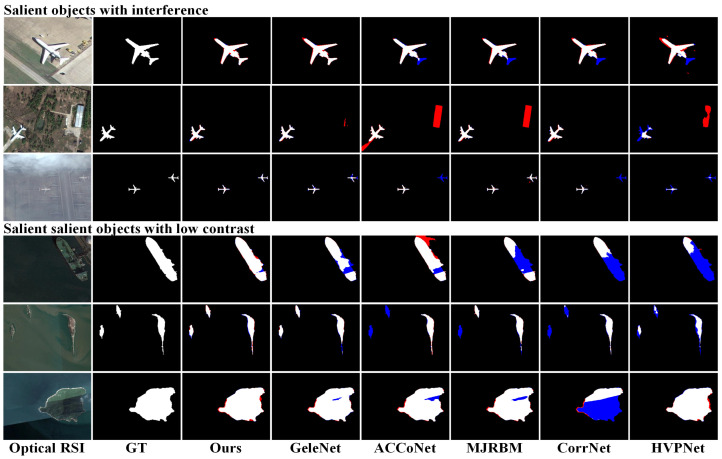
Visual comparison of saliency maps under interference and low-contrast scenes. Red indicates false positives, and blue indicates false negatives.

**Figure 6:**
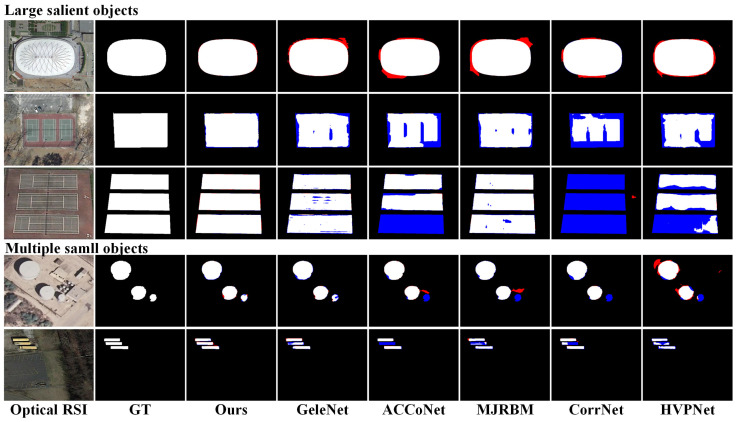
Visual comparison of saliency maps in scenes with a large target and multiple small targets. Red indicates false positives, and blue indicates false negatives.

We present visual comparisons between our approach and some comparative methods in Figure 5 and Figure 6, showing prediction results in different scenarios. In these figures, red annotations indicate false positives, where the model incorrectly identified the background as the target. Blue annotations represent false negatives, where the model incorrectly considered the target as the background. These visual results intuitively illustrate the performance differences among various methods for SOD tasks.

In the first scenario, the target was affected by various factors, as depicted in the first three rows of Figure 5. The images were disrupted by factors such as shadows, complex backgrounds, and dense fog. Only our method accurately detected the target in these scenarios, avoiding false positives and false negatives, demonstrating outstanding anti-interference capabilities and robustness. Some CNN-based methods, such as ACCoNet, although enhancing salient regions by coordinating multi-level features, still suffered from the insufficient feature extraction capability of CNN, resulting in misidentifying the background as the target.

The second scenario involved detecting targets under low-contrast conditions, as illustrated in the last three rows of Figure 5. In this scene, the salient object closely resembled the background in color, leading to confusion between the foreground and background, posing a challenge for SOD. Other methods failed to detect salient regions similar in color to the background. For instance, parts of the boat resembled the color of the water surface, and GeleNet confused the features of these parts, resulting in incomplete boat detection. Our approach produced detection results closest to the ground truth, successfully identifying relatively complete salient targets. This can be attributed to the semantic-aware fusion strategy adopted by SPFM, which selectively enhanced the semantic information of salient targets during the multi-scale feature fusion process while effectively suppressing the influence of background features. This strategy enables the SPFM module to more effectively enhance the discrimination between the foreground and background, thereby assisting the model to more accurately capture the key features of salient targets.

The third scenario involved the detection of large objects, as depicted in the first three rows of Figure 6. In this scene, salient objects occupied a large portion of the entire image, making them easily detectable. However, their backgrounds had complex texture features, which could interfere with the detection performance. The saliency maps generated by other methods exhibited issues such as unclear boundary details and semantic voids within the target. On the contrary, our approach embedded salient semantic information through the SDEM to compensate for missing semantics within large-scale targets. The design of the SDEM addresses the issue of semantic information voids within targets by integrating deep semantic and shallow texture information. Deep semantic information offers an understanding of the overall properties of the targets, while shallow texture information captures subtle features and boundary details of the target.

The fourth scenario involved multiple small objects, as shown in the last three rows of Figure 6. In this scenario, other methods may have missed small targets. Our method, employing the SDEM for multi-scale feature fusion and the SPFM for enhancing local details, accurately segmented all salient objects. This indicates that our approach possesses strong global perception and local detail-capturing capabilities when dealing with the saliency detection task of multiple small targets, thereby enhancing the comprehensiveness and accuracy of SOD.

#### 3.3.2. Quantitative Comparison

We selected 15 methods for quantitative comparison with our approach. All experimental results were provided by the original authors or retrained. Specifically, we retrained six recent SOD methods, including CorrNet, EMFINet, MCCNet, ACCoNet, AESINet, and GeleNet, on the same operational environment, GPU, and default dataset settings. Table 1 and Table 2 report the quantitative comparison results of our approach and other compared methods on MAE, $S_{\alpha }$, ${E_{\xi }}^{adp}$, ${E_{\xi }}^{mean}$, ${E_{\xi }}^{\max}$, ${F_{\beta }}^{adp}$, ${F_{\beta }}^{mean}$, and ${F_{\beta }}^{\max}$ metrics. In these metrics, a lower MAE indicates smaller model errors, while higher values for the other seven metrics suggest better model performance. From the tables, it is evident that our method outperformed other methods on both the ORSSD and EORSSD datasets, demonstrating outstanding performance and generalization capabilities. Specifically, on the ORSSD dataset, our approach surpassed other methods in all metrics except ${E_{\xi }}^{adp}$, ${F_{\beta }}^{adp}$, and ${F_{\beta }}^{mean}$, achieving values of 0.9491, 0.0070, 0.9815, 0.9864, and 0.9253, respectively. On the EORSSD dataset, our approach outperformed the second-ranked method in $S_{\alpha }$ (0.9391 vs. 0.9380), MAE (0.0053 vs. 0.0060), ${E_{\xi }}^{adp}$ (0.9743 vs. 0.9728), ${E_{\xi }}^{mean}$ (0.9784 vs. 0.9740), ${F_{\beta }}^{adp}$ (0.8657 vs. 0.8648), $F_{\beta }^{mean}$ (0.8790 vs. 0.8781), and $F_{\beta }^{max}$ (0.8937 vs. 0.8910).

Additionally, some CNN-based methods, such as CorrNet and ERPNet, adopted different strategies for SOD. CorrNet adopted a coarse-to-fine approach to detect targets, achieving 91.53% $S_{\alpha }$ on the EORSSD. ERPNet provided an additional branch for target edge perception, offering edge cues for SOD and achieving 92.10% $S_{\alpha }$ on the EORSSD. However, constrained by the limited sensitivity of CNN to global information, the overall performance of these methods was unsatisfactory. In contrast, our method enhanced receptive fields through the transformer, obtaining more comprehensive semantic information and achieving a highest $S_{\alpha }$ of 93.91% on EORRSD.

**Figure 7:**
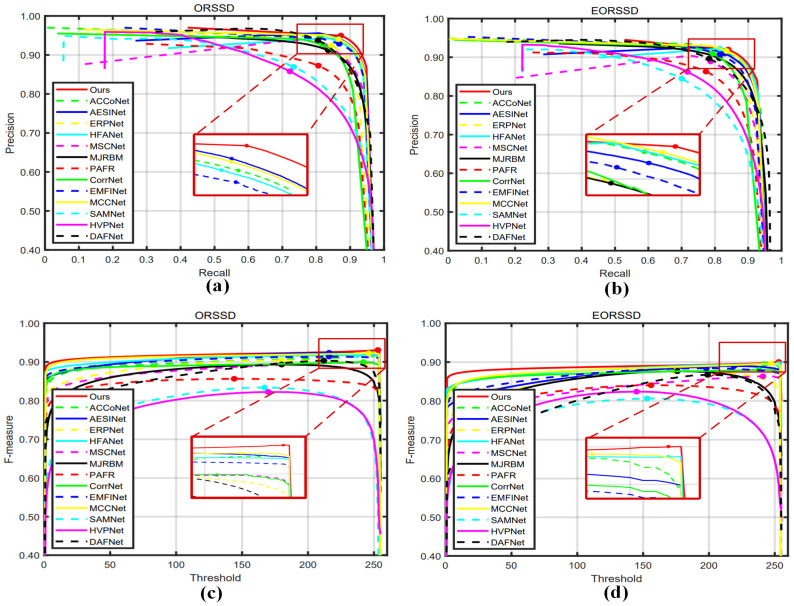
Quantitative comparison of PR curves (**a**,**b**) and F-measure curves (**c**,**d**) for the two datasets.

Figure 7 illustrates the PR curves and F-measure curves for different approaches, serving as an assessment of the overall performance of the models. On both datasets, our method consistently maintained a high detection accuracy as recall increased in the PR curves. Regarding the F-measure curves, our method consistently maintained relatively high F-measure scores across different thresholds. This indicates that our approach had a low false positive rate, further validating the outstanding performance of our method.

### 3.4. Ablation Studies

To validate the effectiveness of each module in our approach, we conducted model analysis and ablation studies on the EORSSD dataset, evaluating the contributions of the SDEM and SPFM modules. We removed the SDEM and SPFM modules from the model, retaining only the UniFormer encoder and a simple decoder as the baseline. The decoder consisted of three up-sampling modules, each composed of two convolutions and one transposed convolution. Skip connections were used to fuse features between the encoder and decoder. Consequently, we designed four different combinations for ablation experiments: (1) baseline, (2) baseline + SDEM, (3) baseline + SPFM, and (4) baseline + SDEM + SPFM. The results of the ablation are shown in Table 3.

From Table 3, we observe that compared to the baseline, the SDEM improved by 0.45%, 0.21%, and 0.35% on metrics $S_{\alpha }$, $E_{\xi }^{max}$, and $F_{\beta }^{max}$, respectively. Similarly, the SPFM enhanced by 0.29%, 0.17%, and 0.17% on metrics $S_{\alpha }$, $E_{\xi }^{max}$, and $F_{\beta }^{max}$, respectively. This directly demonstrates the effectiveness of the two proposed modules. The collaboration between different modules further improved the model’s performance. With all modules included, the complete model surpassed the baseline by 0.24%, 0.32%, and 0.49% on metrics $S_{\alpha }$, $E_{\xi }^{max}$, and $F_{\beta }^{max}$, respectively.

### 3.5. Computational Efficiency Experiment

We evaluated the computational efficiency of various models using two metrics: the number of parameters (Params) and the floating points of operations (FLOPs). Lower Params and FLOPs generally indicate higher computational efficiency. We tested each method with images of the same size (1 × 3 × 256 × 256), and the results are shown in Table 4.

In Table 4, CorrNet was identified as a lightweight network with the fewest parameters, demonstrating excellent computational efficiency but a lower detection accuracy. The methods such as EMFINet, MCCNet, ACCoNet, and ERPNet, which are based on CNN backbones, exhibited poor computational efficiency. Additionally, our Params and FLOPs reached 49.461 M and 11.373 M, respectively, ranking just below GeleNet in terms of computational efficiency. Nevertheless, our model surpassed GeleNet in detection accuracy, making the sacrifice in computational efficiency worthwhile.

## 4. Discussion

This paper presented a new solution for the precise localization and semantic understanding of salient objects in RSI. We explored the potential connections between multi-scale features and fused spatial texture information and abstract semantic expressions. Meanwhile, we realized that the abstract semantic information of salient features might be interfered with by background information during the up-sampling process, thereby enhancing the perception of salient targets through multi-scale features and further filtering out irrelevant information. Our method partially addressed the issues faced by SOD in RSI, such as shadow interference and inter-class feature confusion.

**Figure 8:**
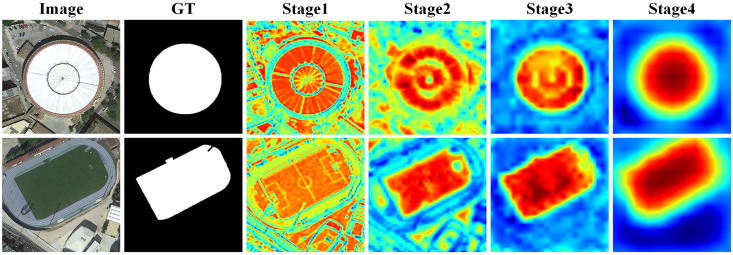
Visual comparison of extracted salient features.

Figure 8 illustrates the process of feature extraction on the input image by GSANet, presenting the actual changes in salient objects at different stages. The attention of the model gradually shifted from global to salient regions, clearly demonstrating the effectiveness of feature extraction. As can be seen in stage 1, the spatial texture details of the input image were clearly visible, while in stage 4, the semantic features of salient objects were prominently evident. This indicates that the shallow network was inclined toward capturing the fine-grained spatial structure of the image but had limited expression for abstract semantics. On the other hand, the deep network focused more on extracting high-level semantic features but lost some detailed spatial information in the process. To fully utilize the information from every stage, a sophisticated fusion of global features and local details is necessary. Thus, we proposed the SDEM and SPFM, which achieved semantic interaction and information fusion across different stages, further retaining the completeness of semantic information and enhancing the global effectiveness of semantic features.

It is clear that as feature extraction progressed, all attention was focused on salient regions. This process clearly demonstrated the effectiveness of our proposed method, which can accurately capture salient features in images based on reliable information, thereby emphasizing its potential advantages in SOD.

## 5. Conclusions

We designed an efficient Global Semantic Perception Aggregation Network, which achieved precise localization and semantic understanding of salient objects in remote sensing images by computing the information entropy of different areas. The information entropy provided a way to measure the complexity and information content of local image regions. The regions with high information entropy often imply the presence of more information in the image and may contain salient objects. By calculating the information entropy of each pixel or region in the image, we can prioritize regions with high information entropy as potential target areas. The advantage of this approach is that it can focus attention on areas that may contain salient objects, thereby improving the efficiency and accuracy of SOD. Therefore, the use of information entropy enables algorithms to better understand the image content and provides important clues for identifying potential salient regions, laying the foundation for further analysis and processing.

GSANet adopted UniFormer to effectively extract local features and global dependencies of salient objects from remote sensing images. Detailed local information preserved more texture details and edge contours, helping the model locate salient objects. Global features assisted the model in understanding the image context information, enhancing the distinction between salient objects and the background. Meanwhile, to better capture global context information and local details, we adopted the SDEM for multi-level feature fusion and semantic interaction. The SDEM searched for high-entropy regions among multi-scale features to effectively aggregate the salient semantic information contained within these regions. Additionally, we also adopted the SPFM to enhance the perceptual capabilities of the model for salient objects during the decoding process. Specifically, the SDEM is a Semantic Detail Embedding Module. By fusing multi-level features, it embedded salient semantic information into low-level features and injected texture details into high-level features. It enabled high-level features to have richer salient information and more clear spatial details, which enhanced perception and understanding of key areas of complex scenes. The SPFM regarded salient semantic features as prior information to compensate for the semantic loss of salient objects during the multi-level feature fusion process, enabling the model to effectively capture and retain crucial salient information. Extensive comparative experiments and ablation studies demonstrated the superiority of our approach, as well as the effectiveness of both the SDEM and SPFM.

The limitation of GSANet is the inability to accurately depict the edge contours of salient objects in some complex scenes, resulting in detected edges that are blurred or fragmented. To address this issue, we will consider utilizing graph convolutional networks [48] in future research to capture the spatial relationships within remote sensing images and further enhance edge features.

## Figures and Tables

**Table 1:** Quantitative comparisons with state-of-the-art SOD methods on ORSSD datasets.

Methods	ORSSD [32]							
	$S_{\alpha }$	MAE	$E_{\xi }^{adp}$	$E_{\xi }^{mean}$	$E_{\xi }^{max}$	$F_{\beta }^{adp}$	$F_{\beta }^{mean}$	$F_{\beta }^{max}$
SAMNet_21_ [38]	0.8761	0.0217	0.8656	0.8818	0.9478	0.6843	0.7531	0.8137
HVPNet_21_ [39]	0.8610	0.0225	0.8471	0.8737	0.9320	0.6726	0.7396	0.7938
DAFNet_21_ [33]	0.9191	0.0113	0.9360	0.9539	0.9771	0.7876	0.8511	0.8928
HFANet_22_ [46]	0.9399	0.0092	0.9722	0.9712	0.9770	0.8819	0.8981	0.9112
MSCNet_22_ [40]	0.9227	0.0129	0.9584	0.9653	0.9754	0.8350	0.8676	0.8927
MJRBM_22_ [41]	0.9204	0.0163	0.9328	0.9415	0.9623	0.8022	0.8566	0.8842
PAFR_22_ [42]	0.8938	0.0211	0.9315	0.9268	0.9467	0.8025	0.8275	0.8438
CorrNet_22_ [13]	0.9201	0.0158	0.9543	0.9487	0.9575	0.8605	0.8717	0.8841
EMFINet_22_ [43]	0.9380	0.0113	0.9637	0.9657	0.9733	0.8664	0.8873	0.9019
MCCNet_22_ [11]	0.9445	0.0091	0.9733	0.9740	0.9805	0.8925	0.9045	0.9177
ACCoNet_23_ [14]	0.9418	0.0095	0.9694	0.9684	0.9754	0.8614	0.8847	0.9112
AESINet_23_ [44]	0.9427	0.0090	0.9704	0.9736	0.9817	0.8667	0.8975	0.9166
ERPNet_23_ [9]	0.9254	0.0135	0.9520	0.8566	0.9710	0.8356	0.8745	0.8974
GeleNet_23_ [47]	0.9451	0.0092	0.9816	0.9799	0.9859	0.9044	0.9123	0.9239
ADSTNet_24_ [45]	0.9379	0.0086	0.9785	0.9740	0.9807	0.8979	0.9042	0.9124
Ours	0.9491	0.0070	0.9807	0.9815	0.9864	0.8994	0.9095	0.9253

The best results are highlighted in red, and the second-best are indicated in blue.

**Table 2:** Quantitative comparisons with state-of-the-art SOD methods on EORSSD datasets.

Methods	EORSSD [33]							
	$S_{\alpha }$	MAE	$E_{\xi }^{adp}$	$E_{\xi }^{mean}$	$E_{\xi }^{max}$	$F_{\beta }^{adp}$	$F_{\beta }^{mean}$	$F_{\beta }^{max}$
SAMNet_21_ [38]	0.8622	0.0132	0.8284	0.8700	0.9421	0.6114	0.7214	0.7813
HVPNet_21_ [39]	0.8734	0.0110	0.8270	0.8721	0.9482	0.6202	0.7377	0.8036
DAFNet_21_ [33]	0.9166	0.0060	0.8443	0.9290	0.9859	0.6423	0.7842	0.8612
HFANet_22_ [46]	0.9380	0.0070	0.9644	0.9679	0.9740	0.8365	0.8681	0.8876
MSCNet_22_ [40]	0.9071	0.0090	0.9329	0.9551	0.9689	0.7553	0.8151	0.8539
MJRBM_22_ [41]	0.9197	0.0099	0.8897	0.9350	0.9646	0.7066	0.8239	0.8656
PAFR_22_ [42]	0.8927	0.0119	0.8959	0.9210	0.9490	0.7123	0.7961	0.8260
CorrNet_22_ [13]	0.9153	0.0097	0.9514	0.9445	0.9553	0.8259	0.8450	0.8597
EMFINet_22_ [43]	0.9284	0.0087	0.9482	0.9542	0.9665	0.8049	0.8494	0.8735
MCCNet_22_ [11]	0.9340	0.0073	0.9609	0.9676	0.9758	0.8302	0.8656	0.8884
ACCoNet_23_ [14]	0.9346	0.0081	0.9559	0.9622	0.9707	0.8248	0.8628	0.8846
AESINet_23_ [44]	0.9362	0.0072	0.9443	0.9618	0.9734	0.7908	0.8507	0.8820
ERPNet_23_ [9]	0.9210	0.0089	0.9228	0.9401	0.9603	0.7554	0.8304	0.8632
GeleNet_23_ [47]	0.9373	0.0075	0.9728	0.9740	0.9810	0.8648	0.8781	0.8910
ADSTNet_24_ [45]	0.9311	0.0065	0.9681	0.9709	0.9769	0.8532	0.8716	0.8804
Ours	0.9391	0.0053	0.9743	0.9784	0.9836	0.8657	0.8790	0.8937

The best results are highlighted in red, and the second-best are indicated in blue.

**Table 3:** Ablation results of evaluating the individual contribution of each module.

No.	Baseline	SDEM	SPFM	EORSSD [33]		
				$S_{\alpha }$	$E_{\xi }^{max}$	$F_{\beta }^{max}$
1	√			0.9367	0.9804	0.8888
2	√	√		**0.9412**	0.9827	0.8923
3	√		√	0.9396	0.9821	0.8905
4	√	√	√	0.9391	**0.9836**	**0.8937**

The best one in each column is shown in bold.

**Table 4:** Analysis of the computational efficiency of various methods.

Methods	Params (M)	FLOPs (G)
CorrNet	**4.086**	21.379
EMFINet	95.086	176
MCCNet	67.652	114
ACCoNet	127	50.422
ERPNet	77.195	171
GeleNet	25.453	**6.43**
Ours	49.461	11.373

The best one in each column is shown in bold.

## Data Availability

Data is contained within the article.
